# Targeting the eCIRP/TREM-1 interaction with a small molecule inhibitor improves cardiac dysfunction in neonatal sepsis

**DOI:** 10.1186/s10020-020-00243-6

**Published:** 2020-12-04

**Authors:** Naomi-Liza Denning, Monowar Aziz, Li Diao, Jose M. Prince, Ping Wang

**Affiliations:** 1 Center for Immunology and Inflammation, The Feinstein Institutes for Medical Research, 350 Community Dr, Manhasset, NY 11030 USA; 2 Elmezzi Graduate School of Molecular Medicine, Manhasset, NY USA; 3 Department of Surgery, Donald and Barbara Zucker School of Medicine At Hofstra/Northwell, Hempstead, NY USA; 4 Division of Pediatric Surgery, Cohen Children’s Medical Center At Hofstra/Northwell, New Hyde Park, NY USA

**Keywords:** Neonatal sepsis, CIRP, TREM-1, Cardiomyocyte, DAMP, Inflammation

## Abstract

**Background:**

Neonatal sepsis and the associated myocardial dysfunction remain a leading cause of infant mortality. Extracellular cold-inducible RNA-binding protein (eCIRP) acts as a ligand of triggering receptor expressed on myeloid cells-1 (TREM-1). M3 is a small CIRP-derived peptide that inhibits the eCIRP/TREM-1 interaction. We hypothesize that the eCIRP/TREM-1 interaction in cardiomyocytes contributes to sepsis-induced cardiac dysfunction in neonatal sepsis, while M3 is cardioprotective.

**Methods:**

Serum was collected from neonates in the Neonatal Intensive Care Unit (NICU). 5–7-day old C57BL/6 mouse pups were used in this study. Primary murine neonatal cardiomyocytes were stimulated with recombinant murine (rm) CIRP with M3. TREM-1 mRNA and supernatant cytokine levels were assayed. Mitochondrial oxidative stress, ROS, and membrane potential were assayed. Neonatal mice were injected with rmCIRP and speckle-tracking echocardiography was conducted to measure cardiac strain. Sepsis was induced by *i.p.* cecal slurry. Mouse pups were treated with M3 or vehicle. After 16 h, echocardiography was performed followed by euthanasia for tissue analysis. A 7-day survival study was conducted.

**Results:**

Serum eCIRP levels were elevated in septic human neonates. rmCIRP stimulation of cardiomyocytes increased TREM-1 gene expression. Stimulation of cardiomyocytes with rmCIRP upregulated TNF-α and IL-6 in the supernatants, while this upregulation was inhibited by M3. Stimulation of cardiomyocytes with rmCIRP resulted in a reduction in mitochondrial membrane potential (MMP) while M3 treatment returned MMP to near baseline. rmCIRP caused mitochondrial calcium overload; this was inhibited by M3. rmCIRP injection impaired longitudinal and radial cardiac strain. Sepsis resulted in cardiac dysfunction with a reduction in cardiac output and left ventricular end diastolic diameter. Both were improved by M3 treatment. Treatment with M3 attenuated serum, cardiac, and pulmonary levels of pro-inflammatory cytokines compared to vehicle-treated septic neonates. M3 dramatically increased sepsis survival.

**Conclusions:**

Inhibition of eCIRP/TREM-1 interaction with M3 is cardioprotective, decreases inflammation, and improves survival in neonatal sepsis.

*Trial registration* Retrospectively registered.

## Introduction

Sepsis is defined as life-threatening organ dysfunction caused by a dysregulated host response to infection (Singer 2016). Neonates are particularly vulnerable to sepsis (Raymond 2017a). Estimates of the global burden of neonatal sepsis vary considerably, but neonatal sepsis is thought to account for approximately 1.4 million neonatal deaths per year (Shane and Stoll 2014; Fleischmann-Struzek 2018). Sepsis-associated cardiac dysfunction in neonates is not as well studied as in older patients. Among adults with sepsis, a recent literature review found that myocardial dysfunction is common with a reported incidence ranging from 10 to 70% (Beesley 2018). This large range is likely due, in part, to the lack of a clinical consensus definition of sepsis-associated cardiac dysfunction (Beesley 2018). Myocardial dysfunction in sepsis considerably worsens outcomes (Beesley 2018; Lv and Wang 2016; Walley 2018). Although there is a relative paucity of data in the neonatal population, pediatric studies have recapitulated these findings (Jain et al. 2018; Weiss 2017; Alzahrani 2017; Abdel-Hady et al. 2012; Boode 2010). In fact, cardiac dysfunction appears to occur more frequently in pediatric and neonatal septic patients as compared to their adult counterparts (Wheeler et al. 2011).

Damage-associated molecular patterns (DAMPs) propagate inflammation in sepsis (Aziz et al. 2013; Denning et al. 2019a) and some have been implicated in increasing sepsis-associated cardiac dysfunction (Lv and Wang 2016; Lu 2020; Hagiwara et al. 2008). Cold-inducible RNA binding-protein (CIRP) is an 18-kDa nuclear RNA chaperone protein that is released extracellularly both passively after necrotic cell death and in response to sepsis, hemorrhage or ischemia–reperfusion injury (Nishiyama 1997; Qiang 2013). We have previously demonstrated that extracellular CIRP (eCIRP) is a DAMP, promoting activation of several cell types including macrophages, lymphocytes, and neutrophils, potentiating cytokine and chemokine production, and propagating formation of neutrophil extracellular traps (NETs) (Qiang 2013; Aziz et al. 2019). eCIRP causes endoplasmic reticulum (ER) stress and Nlrp3 inflammasome activation (Yang et al. 2016). Conversely, CIRP knockout mice are protected from sepsis and acute lung injury (Yang, et al. 2016; Khan et al. 2017). In adult human patients, elevated plasma levels of eCIRP have been correlated with a poor prognosis in patients with sepsis and with lung dysfunction after cardiac surgery (Qiang 2013; Zhou 2015; Chen et al. 2019). However, to our knowledge, eCIRP levels have never been assessed in neonates. Furthermore, the impact of eCIRP on cardiac tissue or cardiac function has never been studied.

Triggering receptor expressed on myeloid cells-1 (TREM-1) is an innate immune pattern recognition receptor expressed primarily on neutrophils and macrophages (Bouchon et al. 2000). We have recently shown that eCIRP is a ligand of TREM-1 (Denning 2020). TREM-1 is an activating, proinflammatory receptor, propagating inflammation independently (Dower et al. 2008), as well as synergistically with the toll-like receptor 4 (TLR4) pathway (Dower et al. 2008; Bouchon et al. 2001). Both eCIRP and TREM-1 are upregulated in sepsis to serve as mediators of inflammation (Qiang 2013; Denning 2020). We recently developed a small eCIRP-derived peptide (RGFFRGG), named M3, that is an antagonist of the eCIRP/TREM-1 interaction. We successfully implemented M3 as a therapeutic agent in adult murine models of sepsis (Denning 2020).

However, the neonatal immune response is dramatically different than its adult counterpart (Raymond 2017b). Neonates rely almost exclusively on innate immunity yet have a relatively downregulated NF-κB pathway compared to adults. Additionally, they have a diminished upregulation of TNF-α related genes, diminished pattern recognition signaling, and impaired neutrophil function (Raymond 2017b; Kan et al. 2016). Furthermore, despite some evidence that soluble TREM-1 may be a biomarker for neonatal sepsis (Qian et al. 2014; Bellos 2018), in neonates with sepsis, TREM-1 upregulation is decreased compared to older infants, children, and adults (Raymond 2017a). Given the differences between the adult and neonatal immune response in sepsis, an investigation into the efficacy of M3 in neonatal sepsis is warranted.

In this study using both in vitro studies and echocardiogram of neonatal mice, we establish the link between eCIRP and cardiac dysfunction in sepsis for the first time. We demonstrate the significance of this finding by reporting elevated levels of eCIRP in septic neonates in the neonatal intensive care unit. We determine the impact of the eCIRP/TREM-1 interaction on systemic inflammation, cardiac function, and survival in a mouse model of neonatal sepsis. Finally, we demonstrate the therapeutic potential of M3 as an inhibitor of eCIRP/TREM-1′s interaction in neonatal sepsis.

## Methods

### Determination of blood CIRP levels in human neonatal samples

After obtaining Institutional Review Board approval (Northwell Health IRB # 19-0511), serum samples were obtained from patients in a neonatal intensive care unit at a tertiary care children’s hospital. Serum samples were collected initially for patient care and the remaining volume in a sample after use for diagnostic testing was used for this study. The medical record was reviewed for demographics and clinical and diagnostic information. Serum was stored at −80 °C until analysis. eCIRP levels in the serum were measured using a human CIRP ELISA kit (American Research Products, Waltham, MA) according to manufacturer’s instructions.

### Experimental animals

House-bred male and female C57BL/6 mice were kept in a 12 h light/dark, temperature-controlled room and fed standard Purina rodent diet. Females were closely observed to ensure an accurate date of birth for all litters. Five to seven-day old neonatal mice were used for all in vivo experiments. Sex determination via external characteristics is difficult to reliably determine at this age and weight (three to four grams). As such, pups were not identified as either male or female and both genders were therefore used in all experiments. Pups remained with their mothers throughout all experimental timeframes and could breastfeed ad libitum. All experimental procedures were performed in accordance with the National Institutes of Health Guidelines for the Care and Use of Laboratory Animals. This study was approved by the Institutional Animal Care and Use Committee of the Feinstein Institutes for Medical Research.

### Murine model of neonatal sepsis

Neonatal sepsis was induced with cecal slurry (CS) as originally developed by Wynn et al. (Wynn 2007) and modified as previously described by us (Denning et al. 2019; Bolognese 2018). Sepsis was induced in neonatal mice via by administration of 0.525 mg/g body weight (BW) intraperitoneal CS. Pups were removed and returned to their cage with their mothers as a group. Sham mice received an intraperitoneal injection of an equivalent volume of 5% dextrose. The use of antibiotics may reduce bacterial load, and therefore inflammation. Given the possibility of misinterpretation of the outcomes of M3 treatment, antibiotics were not used in the sepsis model in order to allow for a rapid, robust, inflammatory response. At 16 h after CS injection, pups were anesthetized using 2.5% isoflurane anesthesia, underwent echocardiogram as described below, and were subsequently euthanized by cardiac puncture. Blood, heart, and lungs were collected. Heart and lungs were immediately flash-frozen in liquid nitrogen. Blood was centrifuged at 1,000 rotations per minute (rpm) for 10 min and serum was collected. Serum, heart, and lungs were then stored at -80 °C until analysis. For the survival study, pups received a diluted dose of CS (0.175 mg/g BW) and were monitored for seven days for survival.

### In vivo administration of rmCIRP and M3

Recombinant murine CIRP (rmCIRP) was produced as previously described (Qiang 2013). rmCIRP at a dose of 10 mg/kg BW or an equivalent volume of normal saline was administered intraperitoneally. M3 (10 mg/kg BW) or vehicle (normal saline) was given *i.p.* at the time of cecal slurry injection (Denning 2020). For the survival study, an additional group of mice received M3 2 h after CS injection.

### Isolation of primary murine neonatal cardiomyocytes

Neonatal cardiomyocytes were isolated from 0 to 2-day old neonatal C57BL/6 mice using the Pierce Primary Cardiomyocyte Isolation kit (ThermoFisher Scientific, Waltham, MA) according to the manufacturer’s instructions. Cells were cultured in DMEM from the kit supplemented with 10% heat-inactivated FBS and 1% penicillin–streptomycin in a humidified incubator with 5% CO_2_ at 37 °C.

### Measurements of reactive oxygen species, mitochondrial depolarization, and mitochondrial calcium levels

Cardiomyocyte and mitochondrial reactive oxygen species (ROS), mitochondrial depolarization, and mitochondrial calcium levels were assayed as described previously by Joseph et al. (Joseph, et al. 2017; Joseph 2016). Cardiomyocytes were plated in 96-well plates. Plated cells were pre-treated with 10 µg/mL M3 peptide or equivalent volume additional media for 20 min. Cells were then stimulated with either PBS as a control or rmCIRP at various doses for 4 h to assess ROS and mitochondrial depolarization or 1.5 h to determine calcium levels. To measure total ROS, cardiomyocytes were loaded with 25 μM 2′,7′-dichlorofluorescin diacetate (DCF) (Sigma-Aldrich, St Louis, MO) for 30 min in the dark. Excess DCF was removed by washing and DCF fluorescence was recorded at excitation/emission wavelengths of 490/530 nm. To measure mitochondrial ROS, MitoSOX Red, a mitochondrial superoxide indicator, (5 uM, ThermoFisher Scientific) was added to cardiomyocytes and incubated in the dark for 30 min. Excess MitoSOX Red was removed, and fluorescence was recorded at excitation/emission wavelengths of 525/620 nm. Tetramethylrhodamine methyl ester (TMRM) (1 nM, ThermoFisher Scientific) was used to assess changes in mitochondrial membrane potential. Cardiomyocytes were stained with TMRM ester for 30 min and florescence was recorded at excitation/emission 540 nm/590 nm. Due to its internal positive charge, TMRM preferentially accumulates inside mitochondria. As the mitochondria depolarizes, less TMRM is trapped. Therefore, the TMRM signal is proportional to the inner membrane potential of the mitochondria (Joseph et al. 2017). Finally, changes in mitochondrial calcium were determined using a 30 min incubation with 10 μM Rhod-2-AM (ThermoFisher Scientific) followed by a 1 h washout- and cytosolic quenching with 0.5 mM manganese (Trevigen, Gaithersburg, MD). Fluorescence was measured at 552 nm (excitation)/581 nm (emission). Fluorescence was measured by using a fluorescence reader (Synergy H1 Hybrid reader, BioTek, Winooski, VT, USA).

### Enzyme-linked immunosorbent assay (ELISA)

Cardiomyocyte supernatant was analyzed by ELISA kits for interleukin (IL)-6 and tumor necrosis factor-α (TNF-α) (BD Biosciences, San Jose, CA) according to the manufacturer’s instructions. Cardiac and lung tissue was crushed in liquid nitrogen, and equal weights of powdered tissues (~ 50 mg) were dissolved in 500 µl of lysis buffer (10 mM Hepes, pH 7.4, 5 mM MgCl_2_, 1 mM DTT, 1% Triton X-100, and 2 mM each of EDTA and EGTA), and subjected to sonication on ice. Protein concentration was determined by the BioRad protein assay reagent (Hercules, CA). Equal amounts of proteins (250–500 µg) were loaded into respective ELISA wells for the assessment of IL-6 and IL-1β (Invitrogen, Carlsbad, CA). Serum was analyzed using a Bio-Plex Pro Mouse Cytokine Th17 Panel A -6-Plex kit (BioRad).

### Real-time quantitative reverse transcription polymerase chain reaction (qRT-PCR)

To examine sepsis-associated lung and cardiac inflammation, the lung and heart mRNA expression of IL-6 and IL-1β were measured. TREM-1 expression in neonatal murine cardiomyocytes was assessed after 24 h of rmCIRP stimulation. Total RNA was extracted from cells and tissue using Trizol reagent (Invitrogen). cDNA was synthesized using MLV reverse transcriptase (Applied Biosystems, Foster City, CA). PCR reactions were carried out in 20 μl of a final volume of 0.08 μM of each forward and reverse primer, cDNA, water, and SYBR Green PCR master mix (Applied Biosystems). Amplification was performed in a Step One Plus real-time PCR machine (Applied Biosystems). Mouse β-actin or GAPDH mRNA was used as an internal control for amplification for lung and cardiac tissue, respectively, and relative gene expression levels were calculated using the ΔΔCT method. Relative expression of mRNA was expressed as fold change in comparison with sham tissues or PBS treated cells.

### Echocardiogram

Two hours after injection of rmCIRP or 16 h after sepsis induction with CS, cardiac function in neonatal mice was assessed by transthoracic echocardiography. Echocardiography was conducted using a 40 MHz center frequency transducer coupled to a Vevo®3100 Imaging System (Fujifilm VisualSonics, Toronto, ON, Canada). Sedation was induced with 2.5% isoflurane and maintained with 0.5–1% isoflurane for the duration of the echocardiogram. Mice were maintained on a heated table during this time. Parasternal long axis views were taken in B and M modes. The B mode imaging provides a two-dimensional view of the heart. The M mode imaging displays one ultrasound line chosen from the two-dimensional image over time (Lindsey et al. 2018). VevoLab (Fujifilm VisualSonics) software was used to determine cardiac parameters. VevoStrain (Fujifilm VisualSonics) software was used to measure myocardial strain and strain rate using speckle-tracking echocardiography (Hoffman 2019).

### Statistical analysis

Data represented in the figures are expressed as mean ± SE. All data has been tested for normality using the Kolmogorov–Smirnov Test of Normality. Normally distributed data was analyzed using the two-tailed Student’s *t* test for two-group comparisons and One-way ANOVA for comparison among multiple groups with the significance between individual groups determined using the Tukey method. Nonparametric data was analyzed using one-way comparison among multiple groups with the Kruskal–Wallis test with a Dunn’s multiple comparison test. Significance was considered for *p* ≤ 0.05 between study groups. Data analyses were carried out using GraphPad Prism graphing and statistical software (GraphPad Software, San Diego, CA).

## Results

### eCIRP levels are increased in neonates with sepsis

**Fig. 1:**
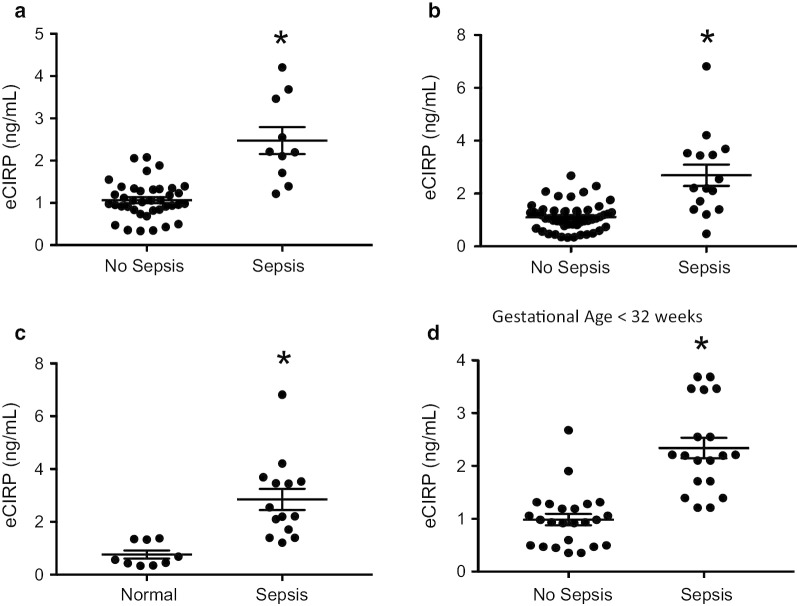
Serum eCIRP levels are elevated in septic neonates. Serum levels of eCIRP were compared in **a** neonates classified as non-septic (N = 39) versus septic (N = 10) according to the electronic medical record. **b** Infants clinically classified as “sepsis rule-out” were divided into the non-septic (N = 52) vs septic (N = 15) cohorts according to duration of antibiotics therapy. **c** Serum levels of eCIRP in full term, well infants (N = 9) were compared to septic neonates (N = 14). **d** Infants with a gestational age < 32 weeks of no sepsis (N = 24) vs sepsis (N = 19) were compared. Data are expressed as means ± SE and compared by two-tailed student’s t test (* p < 0.05 vs no sepsis)

Blood samples were obtained from human neonates in the neonatal intensive care unit (NICU) of a tertiary care children’s hospital. Extracellular CIRP levels in the serum were measured by ELISA. Several comparisons were made between groups. Infants were designated as septic versus non-septic according to review of the electronic medical record (EMR) and documentation on the day of specimen collection. First, eCIRP levels in non-septic infants versus septic infants were compared. Extracellular CIRP levels were significantly elevated in septic neonates (Fig. 1a). Second, because sepsis rule-out is so common in neonates, we included infants in that category in the comparison. Infants that had been clinically classified as “sepsis rule-out” in the EMR on the day of serum collection were dived into two groups. Infants ultimately receiving only 48 h of antibiotics, and thus clinically determined to not have sepsis, were included in the no sepsis group. Infants who received more than 48 h of antibiotics were included in the sepsis group. There was a dramatic difference between the two groups (Fig. 1b). Next, infants with sepsis were compared to a group of infants in the well-baby nursery or with full term infants in the NICU with a short (less than 3 days) length of stay and a non-infectious reason for NICU admission, rendering them likely to be a fairly homogenous, well-baby population (Fig. 1c). Finally, given the large difference in corrected gestational age of infants in the NICU, we compared septic vs non-septic infants with a gestational age at birth of under 32 weeks and again saw large disparities in serum CIRP levels (Fig. 1d). The significant elevations in serum CIRP levels of septic neonates highlights the clinical relevance of the findings in this study.

### M3 attenuates systemic inflammation in murine neonatal sepsis

**Fig. 2:**
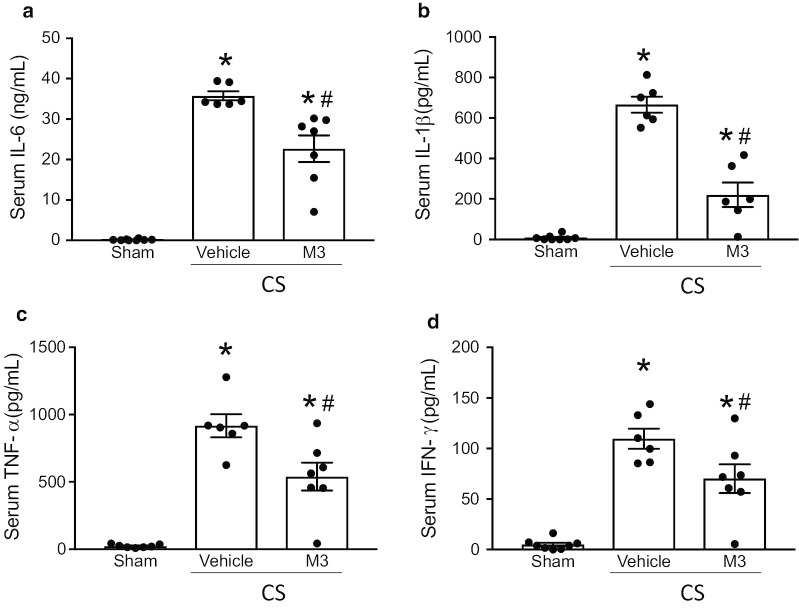
Treatment with M3 reduces proinflammatory cytokines in neonatal sepsis. 16 h after CS injection, serum from sham, vehicle, or M3 treated pups was collected to measure serum cytokines **a** IL-6, **b** IL-1β, **c** TNF-⍺, and **d** IFN-γ by ELISA. Data are expressed as means ± SE (sham = 8, vehicle = 6, and M3 = 7 pups). Multiple groups were compared by one-way ANOVA and Tukey method (*p < 0.05 vs. sham; ^#^p < 0.05 vs. Vehicle)

To evaluate the impact of the eCIRP/TREM-1 interaction in neonatal sepsis, we treated pups with the M3 peptide or normal saline vehicle after sepsis induction with CS. Sixteen h after CS, as a surrogate of systemic inflammation, we measured the serum levels of IL-6, IL-1β, TNF-α, and IFN-γ (Fig. 2). Compared with sham pups, septic pups had significant elevations in serum levels of all four cytokines; IL-6 (Fig. 2a) increased over 200-fold, from an average of 0.17 ng/mL in sham pups to 35.8 ng/mL in vehicle-treated pups. Treatment with M3 dampened this inflammation: M3-treated septic pups had a 36.6% reduction in IL-6 levels to an average of 22.7 ng/mL (Fig. 2a). Similarly, IL-1ß was elevated in septic vehicle-treated pups to an average of 666.0 pg/mL compared to shams’ average level of 8.7 pg/mL. M3 improved this inflammation by 42.7%, resulting in an average level of 381.3 pg/mL (Fig. 2b). Sepsis induction resulted in analogous increases in TNF-α and IFN-γ with increases of 38.7-fold and 22.4-fold, in vehicle-treated septic pups compared to sham pups, respectively. M3 treatment attenuated these levels, by 41.2% and 36.0%, respectively (Figs. 2C-D). These data clearly suggest that M3 plays an anti-inflammatory role by decreasing systemic cytokine levels in neonatal sepsis.

### Treatment with M3 pulmonary and cardiac inflammation in neonatal sepsis

**Fig. 3:**
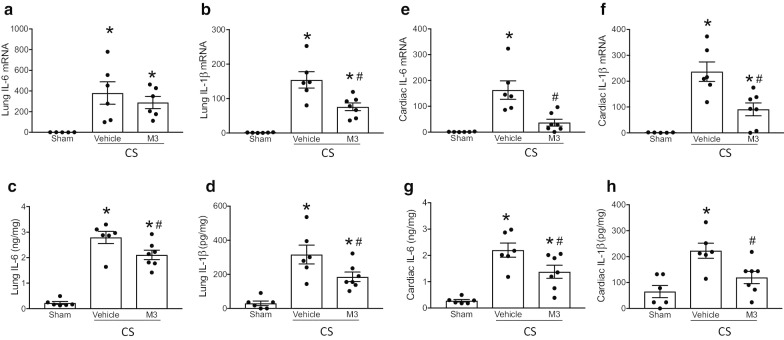
M3 improves pulmonary and cardiac inflammation. 16 h after cecal slurry sepsis induction, mRNA and protein levels of proinflammatory cytokines were measured by RT-PCR and ELISA. Lung levels of IL-6 and IL-1β (**a**, **b**) mRNA and (**c**, **d**) protein were increased by sepsis and reduced in M3 treated pups. Analogously, cardiac IL-6 and IL-1β (**e**, **f**) mRNA and (**g**, **h**) protein were elevated in vehicle treated pups as opposed to M3 treated septic neonates. Data are expressed as means ± SE (sham = 6, vehicle = 6, and M3 = 7 pups). Multiple groups were compared by one-way ANOVA and Tukey method (*p < 0.05 vs. sham; ^#^p < 0.05 vs. Vehicle)

Sepsis-associated acute lung injury is responsible for a large percentage of the morbidity and mortality of intra-abdominal sepsis (Perl et al. 2011). We assessed both mRNA expression and protein levels of neonatal lung tissue 16 h after sepsis induction with cecal slurry. In the lungs, both mRNA and protein levels of the pro-inflammatory cytokines IL-6 and IL-1ß were significantly increased in vehicle treated septic pups compared to sham neonates (Figs. 3A-D). M3 treatment significantly decreased the expression of IL-6 and IL-1ß at mRNA level by 50.7% and 24%, respectively, and at protein level by 41.3% and 24.8%, respectively compared to vehicle-treated samples (Figs. 3A-D). Cytokine production from the heart has been shown to propagate inflammation and worsen cardiac dysfunction (Aoyagi and Matsui 2011; Zhang 2007). Analogously, in the cardiac tissues, both mRNA and protein levels of the pro-inflammatory cytokines IL-6 and IL-1ß were significantly increased in vehicle treated septic pups compared to sham neonates (Fig. 3e-h). M3 treatment significantly decreased the expression of IL-6 and IL-1ß at mRNA level by 77.5% and 61.7%, and at protein level by 37.3% and 46.3%, respectively, compared to vehicle-treated samples (Fig. 3e-h). Thus, M3 decreases proinflammatory cytokine levels in the lungs and cardiac tissues in neonatal septic mice.

### Treatment with M3 improves cardiac function in neonatal sepsis

**Fig. 4:**
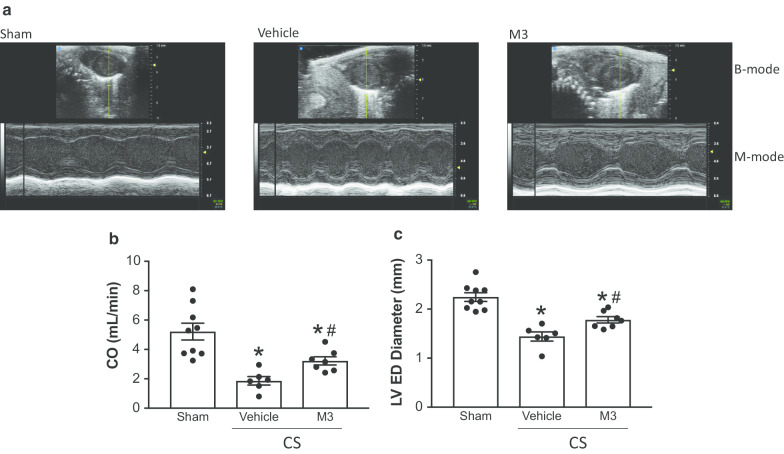
M3 improves cardiac function in neonatal sepsis. 16 h after cecal slurry injection, echocardiogram was performed on mouse pups. **a** Representative still images of B and M-mode echocardiogram findings. The B mode is the top portion of the images, while the M mode is the wave pattern, which is shown at the bottom portion of the images. **b** CO was reduced by sepsis induction while M3 treatment was able to ameliorate some sepsis-induced dysfunction. **c** Left-ventricular end diastolic diameter was reduced in vehicle-treated septic pups as compared to M3-treated pups. Data are expressed as means ± SE (sham = 9, vehicle = 6, and M3 = 7 pups). Multiple groups were compared by one-way ANOVA and Tukey method (*p < 0.05 vs. sham; ^#^p < 0.05 vs. Vehicle)

To determine if M3 improved cardiac function in neonatal sepsis, we performed echocardiogram 16 h after sepsis induction (Fig. 4a, Additional file 1: Video S1). Cardiac output (CO) was dramatically decreased 16 h after sepsis induction. M3 improved CO by 42% (Fig. 4b). Diastolic function was also impaired by sepsis. Left ventricular end diastolic diameter was decreased in vehicle treated mice and improved by 20% in M3 treated pups (Fig. 4c).

### M3 improves survival in neonatal sepsis

**Fig. 5:**
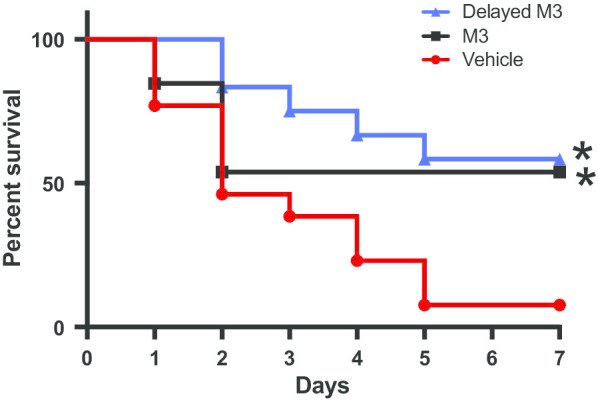
M3 improves survival in neonatal murine sepsis. Mouse pups were injected with a reduced dose of cecal slurry (0.175 mg/g BW) and treated with either M3 or normal saline vehicle. The square black line represents M3 treatment at the time of cecal slurry injection. The triangle blue line represents M3 treatment 2 h after sepsis injection. N = 13 mice per group, *p < 0.05 vs. vehicle, determined by the log-rank test

To verify that the reduction in inflammatory markers and improvement in cardiac function resulted in improved outcomes, we performed a survival study on septic neonates. Pups were injected with a reduced concentration of CS and either M3 or vehicle. Neonatal pups were monitored for seven days. Survival improved from 8% in the vehicle group to 54% in the group of pups who received simultaneous M3 treatment (Fig. 5). To increase the clinical applicability of the model, we also tested the survival in a group of pups exposed to M3 two hours after sepsis induction with CS. This group also had improved survival at 58% (Fig. 5). There was no statistically significant difference in survival between the simultaneous and delayed administration treatment groups. These findings confirm that inhibiting the eCIRP/TREM-1 inhibition improves survival in murine neonatal sepsis.

### Treatment with M3 inhibits rmCIRP-mediated inflammation in neonatal cardiomyocytes

**Fig. 6:**
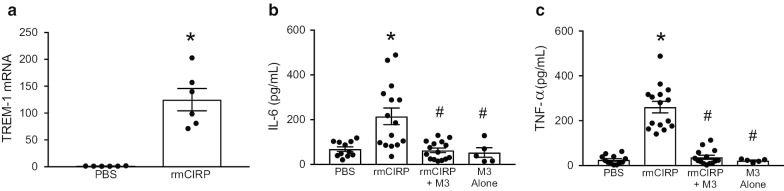
The eCIRP/TREM-1 interaction increases inflammation in cardiomyocytes. Primary murine neonatal cardiomyocytes were isolated and treated with rmCIRP for 24 h. **a** TREM-1 expression was assessed by RT-PCR (N = 6/group). Cardiomyocytes were treated with rmCIRP for 24 h with or without 20-min pretreatment with 10 µg/mL M3. rmCIRP stimulation increased levels of **b** IL-6 and **c** TNF-⍺ in the supernatant. M3 mitigated this inflammatory response. The last column shows M3 treatment alone as a control. Data are result of 2 independent experiments and expressed as means ± SE and compared by Kruskal–Wallis test with Dunn method (*p < 0.05 vs. PBS control and ^#^p < 0.05 vs. rmCIRP-treated cells; PBS = 12, rmCIRP = 15, rmCIRP + M3 = 15, and M3 alone = 5)

Given that M3 was successful in improving cardiac function in neonatal sepsis, we aimed to further identify if this impact was, in part, due to the eCIRP/TREM-1 interaction in cardiomyocytes or if it was merely a reflection of reduced sepsis severity seen with eCIRP inhibition in previous preclinical sepsis models (Qiang 2013; Denning et al. 2019). We first examined the response of neonatal murine cardiomyocytes to rmCIRP. Primary murine neonatal cardiomyocytes were isolated from 1 to 2-day old neonatal mice and plated in 96-well plates. After 24 h of rmCIRP stimulation, cells were lysed and assessed for TREM-1 mRNA expression. rmCIRP increased TREM-1 gene expression by approximately 100-fold compared to PBS-treated cardiomyocytes (Fig. 6a).

To demonstrate that rmCIRP results in cardiac inflammation, primary murine neonatal cardiomyocytes were again isolated and exposed to rmCIRP. rmCIRP stimulation significantly increased levels of IL-6 (Fig. 6b) and TNF-α (Fig. 6c) in the supernatant by 3.1 and 10.3-fold, respectively. However, treatment with rmCIRP after 20 min of pre-treatment with M3 reduced IL-6 and TNF-α levels to near-baseline, by 70.9% and 84.8%, respectively (Figs. 6b, c). Cumulatively, this data indicates that TREM-1 expression increases during inflammatory conditions in cardiac cells and that inhibition of the eCIRP/TREM-1 interaction is anti-inflammatory.

### rmCIRP increases oxidative stress in cardiomyocytes and causes mitochondrial dysfunction

**Fig. 7:**
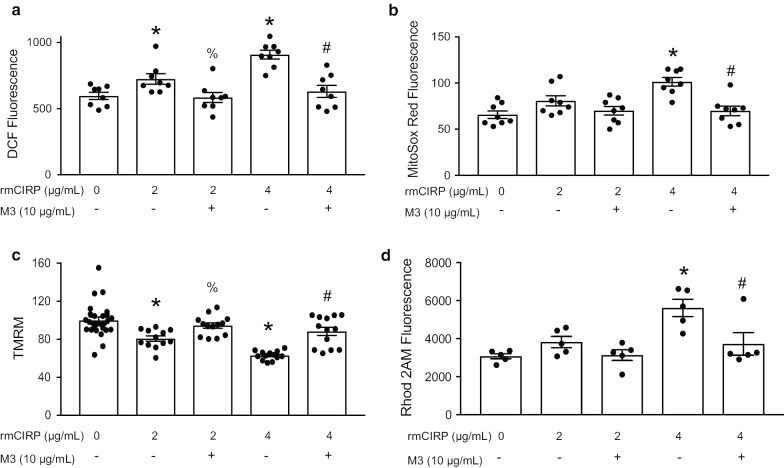
rmCIRP increases oxidative stress in cardiomyocytes and causes mitochondrial dysfunction. Primary murine neonatal cardiomyocytes were isolated. Cardiomyocyte and mitochondrial oxidative stress were quantified using **a** DCF (N = 8/group) and **b** MitoSOX Red fluorescence (N = 8/group). Mitochondrial membrane potential and calcium levels was assessed using **c** Tetramethylrhodamine methyl ester (TMRM) (PBS = 27 and 13 per group for rest) and **d** Rohd2 AM (N = 5/group) fluorescence. After rmCIRP stimulation, there was a significant dose-dependent increase in cardiomyocyte and mitochondrial-specific reactive oxygen species; this was ameliorated by M3 (**a**, **b**). Stimulation of cardiomyocytes with rmCIRP resulted in a reduction in mitochondrial membrane potential while M3 treatment returned membrane potential to near baseline (**c**). rmCIRP caused mitochondrial calcium overload; this was inhibited by M3 (**d**). Data shown are the results of two to three independent experiments. Multiple groups were compared by one-way ANOVA and Tukey method (*p < 0.05 vs. PBS control; % p < 0.05 vs 2 µg/mL rmCIRP, ^#^p < 0.05 vs 4 µg/mL rmCIRP)

To better identify how eCIRP results in cardiac dysfunction in sepsis, we examined cardiac oxidative stress and mitochondrial dysfunction in vitro*.* 2′,7′-dichlorofluorescin diacetate (DCF) fluorescence was used to quantify total cellular ROS in cardiomyocytes after rmCIRP exposure (Fig. 7a). There was a dose dependent increase in ROS after rmCIRP stimulation. M3 was able to significantly inhibit this rmCIRP-mediated increase in ROS (Fig. 7a). To look preferentially at mitochondrial ROS, we used Mitosox Red. We again found a dose dependent increase in ROS after rmCIRP stimulation that was ameliorated by M3 treatment (Fig. 7b). We next quantified the mitochondrial inner membrane potential using TMRM. rmCIRP resulted in a significant reduction in mitochondrial membrane potential in cardiomyocytes which was partially restored by M3 (Fig. 7c). In a homeostatic cell, the mitochondrial inner membrane has a voltage gradient – the interior is negative as compared to the cytosol. rmCIRP caused partial depolarization of the mitochondrial inner membrane potential, an indication of cardiomyocyte mitochondrial dysfunction. The inner membrane potential of a mitochondria can be depolarized by a surplus of mitochondrial calcium uptake (Joseph 2016). As such, we sought to evaluate mitochondrial calcium levels after rmCIRP stimulation. Using Rhod2-AM fluorescence, we found that rmCIRP caused mitochondrial calcium overload, in a dose-dependent manner, which was again mitigated by M3 treatment (Fig. 7d). We conclude from this data that rmCIRP increases oxidative stress in cardiomyocytes and contributes to mitochondrial impairment.

### Global longitudinal and radial strain declines following exposure to rmCIRP

**Fig. 8:**
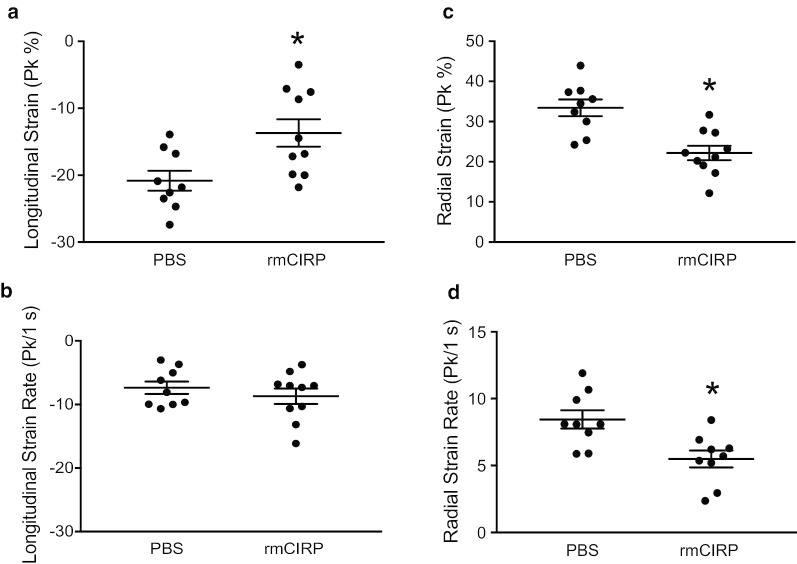
rmCIRP impairs cardiac strain. In vivo rmCIRP exposures causes cardiac dysfunction as evidenced by changes in global longitudinal and radial strain and strain rate. 2 h after rmCIRP injection, mouse pups underwent echocardiogram. B modes images were analyzed using VivoStrain software. rmCIRP injection resulted in impaired **a** longitudinal strain and **b** strain rate as well as impaired **c** radial strain and **d** strain rate. Data are expressed as means ± SE and compared by two-tailed student’s t test (* p < 0.05 vs PBS control; PBS = 9, rmCIRP = 10)

In order to determine if eCIRP was detrimental to cardiac function in vivo*,* 5–7 day old neonatal mouse pups were injected *i.p.* with rmCIRP. Two hours later, using VevoStrain, we assessed radial and longitudinal strain. Myocardial strain allows for quantification of myocardial deformation and has been demonstrated to be an effective assessment of cardiac dysfunction in sepsis (Hoffman 2019). Two hours after rmCIRP injection, pups demonstrated impairment in both longitudinal and radial strain and strain rate (Fig. 8a-d) highlighting the impact of eCIRP on cardiac dysfunction.

## Discussion

The initial response of the innate immune system in neonatal sepsis includes an unregulated, disproportionate release of proinflammatory cytokines in response to PAMPs and DAMPs. This “cytokine storm” can lead to organ dysfunction and death (Raymond 2017b; Khaertynov, et al. 2017; D'Elia et al. 2013). Furthermore, excessive inflammation in the neonatal period has been associated with neurodegenerative effects and cogitative deficiencies (Cardoso 2015; Dubner 2019). Excessive inflammation in the lungs predispose infants to an increased risk of bronchopulmonary dysplasia, while cardiac inflammation has been associated with the development of ventricular failure (Jong et al. 2017; Sun et al. 2017). In the present study of a neonatal murine model of polymicrobial abdominal sepsis, we demonstrated that sepsis treatment with the eCIRP-derived TREM-1 inhibitor M3 reduced systemic, pulmonary, and cardiac inflammation. M3 treatment also improved survival after neonatal sepsis. Although several TREM-1 antagonists are available, most of them were developed and designed from the extracellular TREM-1 sequence and serve as TREM-1 decoy receptors (Pelham et al. 2014). M3 was developed from eCIRP’s sequence to specifically abrogate the eCIRP/TREM-1 ligand/receptor interaction (Denning 2020), while allowing other molecules that regulate inflammation to be un-interrupted. In our previous study, to exclude any off target effects of M3 for inhibiting inflammation in sepsis, we used M3′s scramble peptides which revealed no effects in both in vitro and in vivo conditions (Denning 2020). Moreover, to define the specificity of M3 by using the Biacore and immunofluorescence tools we unequivocally determined that M3 specifically binds to TREM-1 with high affinity (Denning 2020).

The presence of cardiac dysfunction in sepsis can double mortality (Romero-Bermejo et al. 2011). In our study, all neonatal mice who were subjected to sepsis induction via CS demonstrated some degree of cardiac functional impairment as seen on echocardiogram. Unlike adults, sepsis-associated cardiac dysfunction in children is typically a nonhyperdynamic state, with reduced cardiac output and increased systemic vascular resistance (Wynn and Wong 2010). Although neonates can have a more variable response to severe sepsis (Wynn and Wong 2010), we found the anticipated reduction in CO in the neonatal mice. Treatment of septic mouse pups with M3 resulted in an improvement in CO. Maintenance of cardiac output is a key predictor of sepsis survival in infants and children and an important therapeutic target (Weiss 2020; Davis 2017; McGovern and Miletin 2018). Additionally, we found that M3 was able to increase left ventricular end diastolic diameter, a characteristic that has also been associated with increased survival (Huang et al. 2013; Furian 2012). As such, we postulated that the myocardial dysfunction in sepsis was due, in part, to eCIRP.

Although there have been years of research without the identification of a central mechanism for sepsis-associated cardiac dysfunction, several factors have been identified as being contributory. These include upregulation of innate immune receptors in the heart itself, circulating DAMPs and cytokines, altered production of nitric oxide, lack of calcium homeostasis, and oxidative stress (Lv and Wang 2016; Drosatos 2015; Martin 2019). We have previously published ample evidence that eCIRP is released into the circulation in sepsis and results in systemic inflammation (Aziz et al. 2019). In this study, we have demonstrated that circulating eCIRP in sepsis plays a direct role in many of the aforementioned mechanisms of sepsis-associated cardiac dysfunction in neonates.

To demonstrate the direct impact of the eCIRP/TREM-1 interaction on cardiac tissue, primary neonatal cardiomyocytes were stimulated with rmCIRP. rmCIRP was able to upregulate TREM-1 expression in cardiomyocytes. Additionally, stimulated cells increased secretion of both TNF-α and IL-6. Cardiomyocytes are known to develop NF-κβ-dependent inflammation and activation of the transcription of genes involved in producing pro-inflammatory cytokines, including IL-6, TNF-α, and IL-β, during sepsis, which leads to contractile dysfunction in the myocardium (Zhang 2007; Drosatos 2015; Martin, et al. 2016; Merx and Weber 2007; Sun 2015). This inflammation was originally thought to be entirely TLR4 dependent, however evidence is growing that TREM-1 is important in sepsis-associated cardiac inflammation (Boyd et al. 2006; Avlas et al. 2011; Zhou 2014). In addition to promoting inflammation independently (Dower et al. 2008), TREM-1 is known to be synergistic with TLR4 and amplify TLR4-mediated inflammation (Bouchon et al. 2001; Arts 2011; Ornatowska 2007). TREM-1 is also known to proceed through the NF-κβ pathway (Zeng et al. 2007; Gomez-Pina 2012). Thus the protective effects of M3 on cardiac inflammation are likely multifaceted, preventing TREM-1 mediated inflammation and downregulating TREM-1 amplification of TLR4 signaling. Given eCIRP’s interaction with TLR4 to induce inflammation (Qiang 2013), which is also a receptor of LPS, the outcomes of co-stimulation of cells with LPS and eCIRP in terms of producing pro-inflammatory cytokines additively or synergistically are unpredictable and thus may need further study.

Mitochondrial dysfunction is also thought to be a key component of sepsis-associated cardiac impairment (Martin 2019; Lautz and Zingarelli 2019). Sepsis results in partial depolarization of mitochondria and mitochondrial calcium leak, as well as the upregulation of mitochondrial reactive oxygen species, all of which lead to decreased cardiac contractility and dysfunction (Joseph 2016). Oxidative stress as a cause of mitochondrial dysfunction appears to be particularly prominent in neonates (Poggi and Dani 2018). In this study, we have demonstrated that eCIRP is sufficient to increase cardiomyocyte ROS and mitochondrial superoxide, increase calcium influx into mitochondria, and promote depolarization of the mitochondria. Furthermore, we have shown that inhibition of the eCIRP/TREM-1 interaction with the M3 peptide ameliorated these effects. In an observational study of pediatric patients with sepsis, persistent mitochondrial dysfunction was linked to prolonged organ dysfunction (Weiss 2019), therefore the ability to improve mitochondrial function with the M3 peptide is advantageous.

Finally, to verify that eCIRP directly impacts cardiac function in vivo and not just in cell culture, we injected rmCIRP into neonatal mice and measured longitudinal and radial cardiac strain using speckle tracking echocardiography. Cardiac strain is more sensitive than conventional echocardiography for detecting early cardiac dysfunction in sepsis (Hoffman 2019; Li 2014). Myocardial strain can identify sepsis-associated cardiac dysfunction even in the presence of a normal ejection fraction, which can occur with decreased cardiac work and does not accurately reflect cardiac dysfunction (Haileselassie 2017). In a meta-analysis of adult patients with sepsis, increased (i.e., less negative) global longitudinal strain values were associated with mortality; this finding was not seen with ejection fraction – the most commonly used parameter to evaluate adult cardiac function in sepsis (Sanfilippo, et al. 2018). Additionally, changes in myocardial strain have been shown to correlate with the upregulation of inflammatory cytokines and mitochondrial dysfunction in the form of an increased in mitochondrial ROS (Haileselassie 2017). In our study, eCIRP worsened both longitudinal and radial cardiac strain, providing direct evidence that eCIRP causes cardiac dysfunction in neonatal sepsis.

In the current study, we did not assess the bacterial load in the circulation in septic neonates treated with vehicle or M3 despite the fact that each neonate received the same amount of cecal slury. We focused on elucidating the direct anti-inflammatory effect of M3 by targeting the interaction between eCIRP and TREM-1 in immune cells and cardiomyocytes in sepsis. Inflammation has a direct impact on tissue injury and intestinal permeability which causes bacterial translocation in sterile as well as non-sterile inflammation. In our previous study, we demonstrated that M3 treatment protected mice from intestinal ischemia–reperfusion (I/R)-induced injury, (Denning et al. 2020) a sterile injury model which results in increased intestinal tissue injury and permeability. M3 treatment reduced intestinal tissue damage, resulting in less bacterial translocation, and hence less inflammation. Given the M3 treatment led to less inflammation and less tissue damage in murine neonatal sepsis, we may consider that the overall bacterial load in M3 treated mice in neonatal sepsis could be lower than the vehicle-treated mice. For the assessment of surrogates’ markers of sepsis such as the pro-inflammatory cytokines and organ injury markers we only utilized a simultaneous treatment approach with M3 while inducing sepsis by cecal slurry. However, to assess survival, in order to better mimic clinical conditions, along with the simultaneous treatment strategy, we also utilized a post-treatment strategy. In both strategies, we found a statistically significant improvement in survival in neonatal sepsis. As improvements in survival in the simultaneous treatment group were concordant with reduced inflammatory markers in the short term experiments, we anticipate that mice treated with M3 in a delayed manner would also have improved inflammatory markers, as their survival was also improved.

In an attempt to verify the clinical applicability of these findings, we included in our study an analysis of serum eCIRP levels in human neonates. For adult patients, organ dysfunction can be represented by an increase in the Sequential [Sepsis-related] Organ Failure Assessment (SOFA) score of ≥ 2 points, which is associated with an in-hospital mortality greater than 10% (Singer 2016). Nonetheless, SOFA score is not applicable to neonates. The lack of consensus definition for neonatal sepsis is well recognized and unfortunately a limitation of the human data for this manuscript. The patient was classified as septic versus non-septic at the discretion of the treating neonatologist as determined by review of the medical record. At our institution, daily progress notes in the neonatal intensive care unit are largely standardized and typically include a statement on the presence or absence of sepsis. Serum levels of eCIRP have been shown to correlate with severity of illness and survival in adult patients with sepsis (Zhou 2015). However, plasma levels of DAMPs and cytokines are known to vary with age, and a level that is prognostic in one age group does not necessarily extrapolate to another (Decker et al. 2017; Caro 2016). In our study, higher serum eCIRP levels were found in infants with sepsis than compared to non-septic neonates in the NICU. Recognizing that NICU infants are a heterogenous group, many of whom may have chronic inflammation due to noninfectious comorbidities, we further compared septic neonates with a small group of well infants. Differences between these two groups were even more apparent.

In this study, M3 treatment alone did not show any increase in the production of pro-inflammatory cytokines by the primary cardiomyocytes isolated from the neonatal mice. In addition, cell proliferation assays in the presence of 10 times higher than the doses used in vitro in this study of M3 demonstrated no toxicity (Denning 2020). In line with these in vitro data our previous study mentioned that M3 treatment did not demonstrate immunogenicity or tissue injury, as demonstrated by cytokine levels, organ injury markers, or histological analysis in adult mice (Denning 2020). However, prior to use of M3 in large animal models or human clinical trials, further studies on the stability, half-life, and safety would be required. Our study is limited by the lack of invasive cardiac monitoring in the neonatal mice, a limitation due to technical difficulty given their small size. Additionally, we demonstrated the improvement in inflammation and cardiac function when M3 was given concurrently with CS injection. In the clinical setting, it can be difficult to detect the onset of neonatal sepsis. We attempted to mitigate this limitation by providing a survival curve with a delayed M3 administration.

## Conclusion

In conclusion, in this study, we used both in vitro and in vivo data to demonstrate that eCIRP is responsible for some of the cardiac dysfunction seen in septic shock. We further demonstrate that M3, an eCIRP-derived TREM-1 inhibitor, decreases systemic, pulmonary, and cardiac inflammation, improves cardiac dysfunction, and increases survival in a murine model of neonatal sepsis. Pharmacologic inhibition of the eCIRP/TREM-1 interaction is a new potential therapeutic strategy in the treatment of neonatal sepsis.

## Supplementary information

**Additional file 1: Video S1.** Echocardiogram of Septic Mice Representative videos of the echocardiogram of sham, vehicle-treated, and M3-treated neonatal mice 16 h after CS administration.

## Data Availability

All data were presented in the manuscript and in the supplemental section and these are readily available to the readers.

## Abbreviations

eCIRP: Extracellular cold-inducible RNA-binding protein
CIRP: Cold-inducible RNA-binding protein
rmCIRP: Recombinant murine cold-inducible RNA-binding protein
TREM-1: Triggering receptor expressed on myeloid cells-1
MMP: Mitochondrial membrane potential
DAMP: Damage-associated molecular pattern
NETs: Neutrophil extracellular traps
ER: Endoplasmic reticulum
CS: Cecal slurry
BW: Body weight
DCF: Dichlorofluorescin diacetate
TMRM: Tetramethylrhodamine methyl ester
NICU: Neonatal intensive care unit
CO: Cardiac output
LVED Diameter: Left ventricular end diastolic diameter
EMR: Electronic medical record
i.p.: Intraperitoneal
